# Type-1 Collagen differentially alters β-catenin accumulation in primary Dupuytren's Disease cord and adjacent palmar fascia cells

**DOI:** 10.1186/1471-2474-10-72

**Published:** 2009-06-19

**Authors:** Linda Vi, Anna Njarlangattil, Yan Wu, Bing Siang Gan, David B O'Gorman

**Affiliations:** 1 Cell and Molecular Biology Laboratory, Hand and Upper Limb Centre, Lawson Health Research Institute, London, Canada; 2 Department of Surgery, University of Western Ontario, London, Canada; 3 Department of Physiology and Pharmacology, University of Western Ontario, London, Canada; 4 Department of Medical Biophysics, University of Western Ontario, London, Canada; 5 Department of Biochemistry, University of Western Ontario, London, Canada

## Abstract

**Background:**

Dupuytren's Disease (DD) is a debilitating contractile fibrosis of the palmar fascia characterised by excess collagen deposition, contractile myofibroblast development, increased Transforming Growth Factor-β levels and β-catenin accumulation. The aim of this study was to determine if a collagen-enriched environment, similar to *in vivo *conditions, altered β-catenin accumulation by primary DD cells in the presence or absence of Transforming Growth Factor-β.

**Methods:**

Primary DD and patient matched, phenotypically normal palmar fascia (PF) cells were cultured in the presence or absence of type-1 collagen and Transforming Growth Factor-β1. β-catenin and α-smooth muscle actin levels were assessed by western immunoblotting and immunofluorescence microscopy.

**Results:**

DD cells display a rapid depletion of cellular β-catenin not evident in patient-matched PF cells. This effect was not evident in either cell type when cultured in the absence of type-1 collagen. Exogenous addition of Transforming Growth Factor-β1 to DD cells in collagen culture negates the loss of β-catenin accumulation. Transforming Growth Factor-β1-induced α-smooth muscle actin, a marker of myofibroblast differentiation, is attenuated by the inclusion of type-1 collagen in cultures of DD and PF cells.

**Conclusion:**

Our findings implicate type-1 collagen as a previously unrecognized regulator of β-catenin accumulation and a modifier of TGF-β1 signaling specifically in primary DD cells. These data have implications for current treatment modalities as well as the design of *in vitro *models for research into the molecular mechanisms of DD.

## Background

Dupuytren's contracture, or Dupuytren's Disease, (DD) [1-3] is a common, benign palmar fibromatosis of unknown etiology that results in finger contracture and loss of hand function. The most widely accepted treatment is surgical resection of the disease cord, an approach associated with prolonged post-operative rehabilitation and high recurrence rates [4,5]. Recently, minimally invasive treatment alternatives such as clostridial collagenase injection [6,7] and needle aponeurotomy [8-10] have gained popularity. While these approaches require relatively little post-treatment rehabilitation, their long-term efficacy and disease recurrence rates relative to fasciectomy are yet to be clearly established.

We and others have identified dysregulated genes in primary cultures of DD cells [11] or DD cord and nodule tissue [12-14]. Many of these gene transcripts encode extracellular matrix (ECM)-associated proteins, including several types of collagen. Biochemical analyses of DD cord demonstrate the abundance of type I and III collagen [15-17] with type IV and other collagens present to a lesser extent [18].

As with many fibroproliferative conditions, DD is associated with alterations in Transforming Growth Factor (TGF)-β signaling pathways [19-25] and this cytokine promotes both collagen production and contractile myofibroblast development in this disease [26,27]. TGF-β1 has been shown to stimulate fibroblast proliferation by inducing β-catenin accumulation and transactivation of the Tcf/Lef transcription complex during normal and abnormal cutaneous wound repair [28-30]. Primary DD fibroblasts are reported to have an enhanced sensitivity to TGF-β1 signaling [31] and we have previously documented that surgically resected DD cord contains elevated levels of β-catenin [32], implying that TGF-β induced β-catenin accumulation may promote fibroblast proliferation in DD. We have also previously demonstrated that β-catenin levels are altered by isometric tension and ECM-cellular interactions in three dimensional collagen culture in DD cells relative to palmar fascia (PF) cells derived from the same patients [32-34]. These findings suggest that isometric tension during FPCL contraction, collagen interactions or both differentially regulate β-catenin accumulation in these cultures and that changes in β-catenin levels may also be a component of increased contractility that DD cells display relative to patient matched PF cells.

To discern the contribution of collagen to the regulation of cellular β-catenin levels, we have cultured DD and PF cells on type-1 collagen-coated trays in the absence of three-dimensional contraction with or without exogenous addition of TGF-β1. We hypothesized that the presence of type-1 collagen in DD cell cultures, designed to more closely recapitulate *in vivo *conditions, would differentially regulate the responsiveness of DD and/or PF cells to environmental stimuli such as TGF-β1 resulting in changes in β-catenin accumulation.

## Methods

### Clinical Specimen collection

Surgically resected Dupuytren's Disease (DD) cord and small samples of phenotypically normal palmar fascia tissue (PF) were collected from patients undergoing primary surgical resection of DD at the Hand and Upper Limb Centre, London, Ontario. None of these patients were being treated for recurrent disease. All subjects provided written informed consent under institutional review board approval and specimens were collected with the approval of the University of Western Ontario Research Ethics Board for Health Sciences Research involving Human Subjects (HSREB protocol # 08222E).

### Primary cell culture

Primary cells were isolated from surgically resected DD cord and adjacent, phenotypically normal palmar fascia using routine tissue culture practice as previously described [34]. In brief, tissues were aseptically dissected and pressed onto 100 mm culture dishes in α-MEM-medium supplemented with 10% fetal bovine serum (FBS, Invitrogen Corporation, Carlsbad, CA) and 1% antibiotic-antimycotic solution (Sigma-Aldrich, St Louis, MO). Once cellular outgrowths from the tissue fragments were evident, the cells are passaged by routine trypinization. Primary cells isolated by this procedure invariably display a fibroblastic morphology. Six DD cord-derived cell cultures and six patient-matched PF-derived cell cultures were used for these experiments.

For *in vitro *culture on collagen, collagen fibers were mechanically extracted from rat tail tendons (adapted from [35]), placed under UV light overnight and then incubated in sterile acetic acid, with mechanical stirring for 7 days at 4°C. Undissolved collagen fibers were removed by centrifugation at 10,000 × g at 4°C for two hours. Collagen concentration was determined using the Sircol Collagen Quantification assay (Biocolor Ltd., Carrickfergus, UK). For tissue culture, collagen monolayers were cast in 6-well tissue culture trays with each well containing 800 μl collagen and 200 μl of the neutralization solution (2 parts 0.34 N NaOH and 3 parts 10× Waymouth media) to a final concentration of 1.9 mg/ml. Following collagen polymerization, primary cells were added in α-MEM, 10% FBS and 1% antibiotic-antimycotic solution at 37°C in 5% CO_2 _for 72 hours. After 72 hours, media was aspirated from each well and cells were treated as described in the text.

Recombinant Transforming Growth Factor (TGF)β-1 (R&D Systems, Minneapolis, MN) was included in cell cultures as described in the text.

### Immunoblotting

Cells cultured on collagen coated trays were washed with PBS prior to treatment with Collagenase XI (Sigma-Aldrich, St. Louis, MO) to dislodge the adherent cells. The cells were pelleted by centrifugation and cell lysates were prepared in PhosphoSafe protein Extraction Buffer in accordance with the manufacturer's instruction. Cell grown on tissue culture plastic trays were lysed directly in PhosphoSafe protein Extraction Buffer. After centrifuging the extracts to remove insoluble material, equivalent protein quantities were determined by BCA analysis, subjected to immunoblotting and probed with antibodies against β-catenin (BD Biosciences, Mississauga ON), α smooth muscle actin (AbCam, Cambridge MA) and β Actin (Labvision, Fremont, CA). Immunoblot analysis was carried out using standard procedures and immunoreactivity was visualized using Enhanced Chemiluminescence (ECL). A minimum of three and a maximum of six DD and patient-matched PF cell cultures were used for these experiments. All immunoblots were repeated a minimum of three times and representative blots are shown. Intensity of signals relative to β-actin controls were calculated by densitometry using ImageJ software  and averages of arbitrary densitometry units and standard error of data obtained from at least 3 independent experiments were calculated. Paired t-tests were used to assess the significance of differences between groups. Results were deemed significant when p < 0.05.

### Immunofluorescence microscopy

Cells cultured on collagen were fixed with 4% paraformaldehyde in PBS for 20 min and permeabilized with 0.1% Triton X-100 in PBS for 15 min. The preparations were then blocked with 5% non-fat skim milk for 1 hr in PBS. Cells were stained for DNA (DAPI) actin stress fibers (Alexa 488 phallodin, Molecular Probes, Eugene OR), and β-catenin (TRITC-conjugated anti-β-catenin, BD Biosciences, Mississauga ON). All images were captured using a CoolSnap digital camera attached to an IX81 Olympus Inverted Microscope. Three DD and three patient-matched PF cell cultures were used for these experiments. All immunofluorescence studies were repeated three times and representative images are shown.

## Results

### Collagen culture differentially regulates β-catenin accumulation in primary DD and PF cells

**Figure 1:**
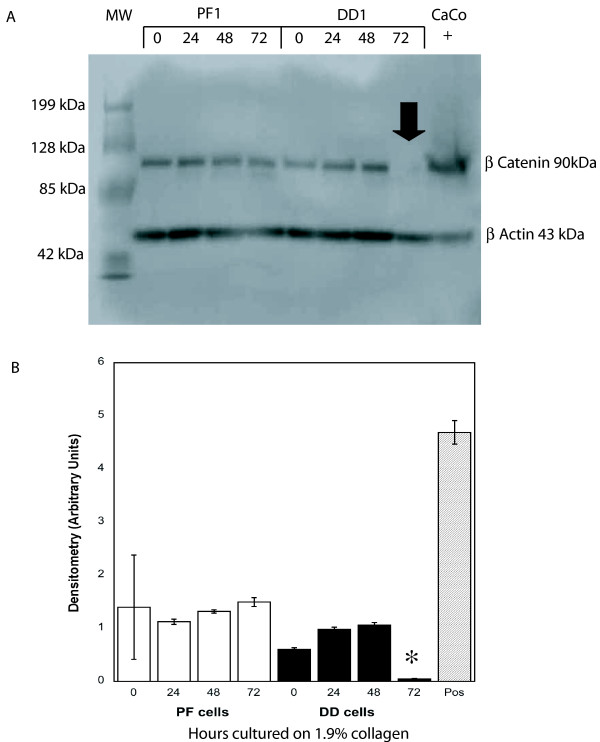
**Type 1 collagen culture effects on β-catenin accumulation in DD and PF-cells**. **A **Representative western immunoblots of PF cells (PF1) and DD cells (PD1) cultured on type 1 collagen (1.9 mg/ml) for 0, 24, 48 and 72 hours prior to cell lysis and 8% PAGE, probed for β-catenin. Lysate from CaCo cells, which express high levels of β-catenin, was included as a positive control. β-actin levels were assessed by immunoblotting to confirm equal total protein loading. As indicated, (arrow) a decrease in β-catenin levels is evident in DD cells cultured on type 1 collagen for 72 hours. **B **Pooled densitometric analysis of triplicate analyses of the samples assessed in A normalized to β-actin. A significant decrease in band density indicating decreased β-catenin levels is evident in DD cells cultured on type 1 collagen for 72 hours relative to 0 hour cultures (*, p < 0.05).

**Figure 2:**
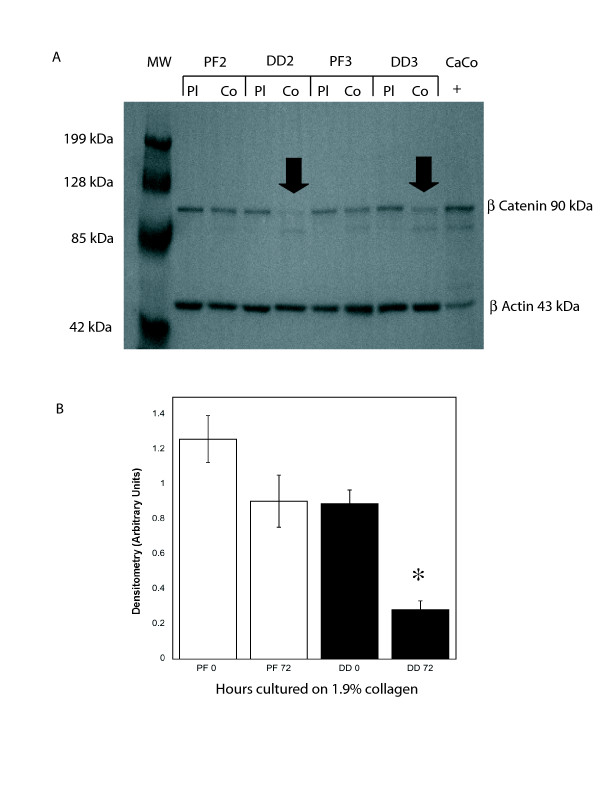
**Type 1 collagen culture effects on β-catenin accumulation are consistent between patient samples**. **A **Representative western immunoblot analysis of additional patient matched sets of PF cells (PF2 and PF3) and DD cells (PD2 and PD3) cultured for 72 hours on either tissue culture plastic (Plc) or type 1 collagen (Col) and probed for β-catenin. β-actin levels were assessed to confirm equal total protein loading. As indicated, (arrows) a decrease in β-catenin levels is evident in DD cells cultured on collagen for 72 hours. **B **Pooled densitometric analysis of 6 patient matched PF and DD cell samples cultured for 72 hours on type 1 collagen normalized to β-actin. As shown, a consistent, significant decrease in band density indicating decreased β-catenin levels is evident in DD cells cultured on type 1 collagen coated dishes for 72 hours relative to 0 hour cultures (*, p < 0.05).

**Figure 3:**
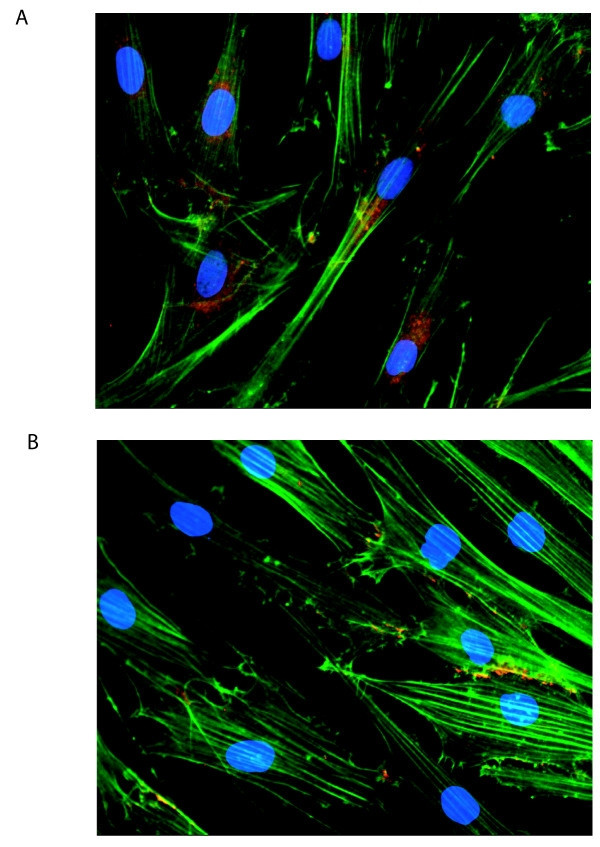
**Type 1 collagen culture effects on β-catenin accumulation in DD and PF-cells**. PF cells (A) and DD cells (B) were cultured on Type 1 collagen coated slides (1.9 mg/ml) for 72 hours. Cells were fixed and stained with Oregon green phalloidin (green) and DAPI (blue) to label filamentous actin and the nucleus, respectively. β-catenin was detected using primary beta-catenin and TRITC-conjugated secondary antibodies (orange). As shown, PF cells consistently displayed readily detectable peri-nuclear staining as well as diffuse staining throughout the cytoplasm after three days on collagen culture. In contrast, DD cells displayed distinct β-catenin staining at the periphery of some cells adjacent to the cell membrane, with little or no β-catenin evident in the cytoplasm after three days on collagen culture.

To distinguish the effects of collagen culture on β-catenin levels from those induced by isometric tension in three dimensional culture, primary DD and PF cells were cultured on collagen coated dishes (1.9 mg/ml) in α-MEM-medium supplemented with 10% FBS for 0, 24, 48 and 72 hours, lysed and β-catenin protein levels were assessed by western blotting. As shown in Figure. 1A and 1B, DD cells display a significant decrease in β-catenin levels at 72 hrs not evident in PF cells derived from the same patient. To confirm that this effect was not specific to these particular cultures and the contribution of collagen to this phenotype, five additional patient-matched sets of DD and PF cells were cultured for 72 hours on either tissue culture plastic or collagen. The decrease in β-catenin levels at 72 hrs was evident in the DD cells from all additional patients tested (two representative sets of patient-derived samples are shown in Figure. 2A, densitometric analysis in Figure. 2B) and collagen culture was required for this effect to be evident. To further confirm this phenotype, three matched sets of DD and PF cells were cultured on collagen coated slides for 72 hours, fixed and stained with Oregon green phalloidin and DAPI to label filamentous actin and nuclei, respectively. β-catenin was detected using primary β-catenin and TRITC-conjugated secondary antibodies. As shown in the representative immunofluorescence image in Figure. 3, PF cells exhibit readily detectable cytoplasmic β-catenin after 72 hours of culture on collagen (Figure. 3A) while cytoplasmic β-catenin was at low to undetectable levels in DD cells under identical culture conditions (Figure. 3B).

### Collagen culture modifies TGFβ-1-induced β-catenin accumulation and α-smooth muscle actin expression in DD and PF cells

**Figure 4:**
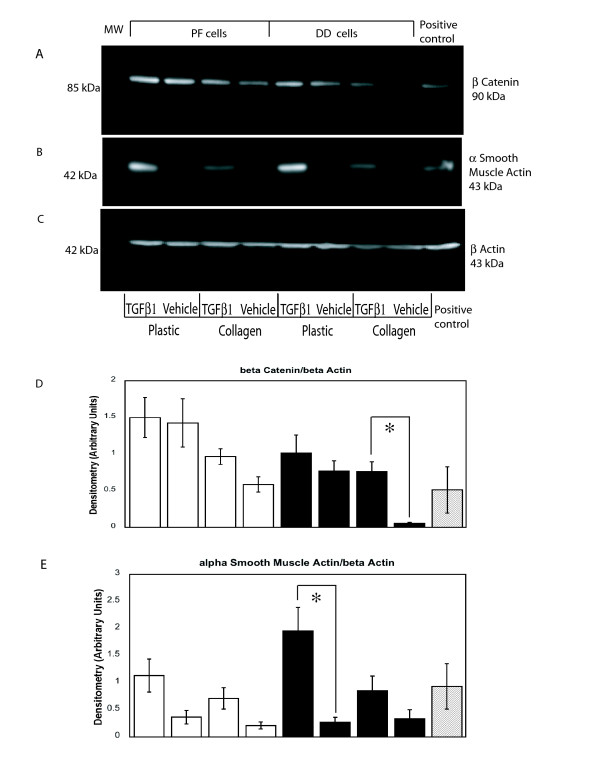
**Type 1 collagen culture effects on TGF-β1 induced α-smooth muscle actin levels and β-catenin accumulation in DD and PF-cells**. Representative western immunoblot analysis of PF and DD cells cultured on tissue culture plastic ("Plastic") or type 1 collagen ("Collagen"), treated with TGFβ-1 or vehicle and assessed for α smooth muscle actin (**A**) and β-catenin (**B**) protein levels. β-actin levels (**C**) were assessed to confirm equal loading. Pooled densitometric analysis of α smooth muscle actin (**D**) and β-catenin (**E**) band density normalized to β-actin band density from triplicate experiments are shown. A significant increase in β-catenin band density after TGFβ-1 treatment of DD cells cultured on collagen (*, p < 0.05) is evident relative to treated cells on plastic while the significant increase in α smooth muscle actin band density after TGFβ-1 treatment of DD cells growing on plastic (*, p < 0.05) is attenuated by collagen culture.

To determine if exogenous TGFβ-1 could rescue this loss of cytoplasmic β-catenin accumulation, cells were cultured in α-MEM-medium supplemented with 10% FBS for 72 hours in the presence or absence of collagen, then changed to α-MEM-medium supplemented with 2% FBS and treated for 72 hours with 12.5 ng/ml TGFβ-1 or vehicle. Our preliminary data (not shown) confirmed the findings of Wong and Mudera [36] that 12.5 ng/ml of TGFβ-1 optimally induced contraction and α-SMA production by DD cells. As shown in Figure. 4A, exogenous addition of TGFβ-1 enhanced cytoplasmic β-catenin accumulation in DD cells cultured on collagen while levels were comparatively unchanged by TGFβ-1 treatment of PF cells under identical conditions. In the absence of collagen, TGFβ-1 treatment did not alter significantly β-catenin levels in either DD or PF cells. In contrast, TGFβ-1 treatment resulted in strongly enhanced levels of α-SMA in the absence of collagen and the addition of collagen markedly attenuated this effect in both DD and PF cells (Figure 4B). These changes, evident by visual inspection, were confirmed by densitometric analysis (Figure. 4D, E).

## Discussion

The data reported here emphasize the importance of recognizing the disease environment as a major contributor to changes in gene expression and cellular phenotype. We have utilized *in vitro *models that mimic one aspect of the *in vivo *DD environment, excess collagen deposition, and show that this single addition results in rapid depletion of cytoplasmic b-catenin levels and markedly alters DD cell responses to TGFβ-1.

These studies utilized primary fibroblastic cells derived from surgically resected DD cord tissue and patient-matched, phenotypically normal palmar fascia. While several studies have indicated that nodular tissue is inherently more "biologically active" than cord tissue *in vivo *[14,31,37,38], many have also noted that primary cells derived from either of these structures are very similar in appearance and responsiveness to TGFβ [31,37]. These observations correlate with more recent studies indicating that cells derived from primary nodule and cord have very similar gene expression profiles [39]. As Dupuytren's Disease cords are surgically resected much more frequently than nodules, we have utilized these abundant and biologically useful tissues to allow us to compare multiple matched pairs of primary cells in this study.

The collagen used for these experiments, extracted from rat tail tendon, is primarily type 1 [40,41] while DD cord has been reported to contain a mixture of type I and III collagens [15-17] with type IV and other collagens present to a lesser extent [18]. DD cord collagen is also reported to feature increased levels of hydroxylysine and reducible cross-links, biochemical changes associated with connective-tissue repair [42]. As collagen type III is an α1(III)3 homotrimer, while type I collagen is usually either an α 1(I)2 and α 2(I) heterotrimer or an α 1(I)3 homotrimer [43], it is likely that our culture system, while closer to *in vivo *conditions than plastic culture trays, does not recapitulate some aspects of the DD cord environment. Future studies will include determining if addition of type-III and other collagens into our *in vitro *model system further modifies any of the disease-specific responses identified in this report.

Previous reports have indicated that primary DD cells are more sensitive to a subset of cytokines including TGFβ-1 than phenotypically normal control cells [31,44]. We have shown that a collagen-rich environment alters the responses of DD cells to exogenous TGFβ-1, a well recognized cytokine component of DD cord *in vivo *[26,27,45]. Contractile fibroblasts can activate latent TGFβ in the ECM [46], suggesting the possibility that simultaneous activation of mechano-receptors and latent, ECM-associated TGFβ may induce contractility in a positive feed-back loop in this fibrosis. It is possible that the altered induction of α-SMA and β-catenin by TGFβ-1 treatment reported here may reflect changes in substrate stiffness between tissue-culture plastic and collagen surfaces, thereby altering concurrent mechano-receptor and TGFβ receptor activation.

TGFβ-1 signaling has been reported to utilize the combinatorial effects of other signaling molecules to direct fibroblasts toward either a proliferative or a contractile phenotype [47]. In our experimental system, type-1 collagen was shown to modify cellular responses to exogenous TGFβ-1, specifically increasing β-catenin accumulation in DD cells and attenuating the increase in α-SMA levels in both DD and PF cells. Overall, these data are consistent with type-1 collagen modifying TGFβ-1 induced proliferation and contractile myofibroblast differentiation in DD.

It is currently unclear to what extent collagen acts to sequester TGFβ-1, thereby decreasing its bioavailability, or modifies cellular signaling cascades resulting in altered cellular responses. Integrins are the primary mediators of signals from the extra-cellular matrix that affect cellular gene expression or changes in morphology [48,49]. We speculate that novel combinations of integrins in DD cells may mediate the increased sensitivity of DD cells to their collagen-enriched environment. TGFβ1 can induce α5β1 expression in other cell types [50,51] and DD cord myofibroblasts are reported to express higher levels of α5β1 integrins than cells in surrounding tissues [52,53]. Type 1 collagen/β1 integrin interactions have been reported to alter cytoplasmic β-catenin accumulation in tumor cells through tyrosine kinase-mediated disruption of adherens junctions [54]. We have previously reported tyrosine phosphorylated β-catenin accumulation in collagen-rich DD cord tissue [32], indicating that tyrosine kinase-mediated adherens junction disruption may contribute to the cytoplasmic β-catenin evident in DD tissue. Cytoplasmic β-catenin levels are regulated through serine-9 phosphorylation and inactivation of Glycogen Synthase kinase (GSK)3β in the canonical pathway rather than through changes in transcriptional activity of *CTNNB1*, the gene encoding β-catenin [19]. Studies are currently underway to assess both tyrosine phosphorylated β-catenin levels and Glycogen Synthase kinase (GSK)3β activity in DD and PF cells grown on collagen in the presence or absence of TGFβ1 to clarify the source(s) of β-catenin in DD.

Current treatment for DD includes surgical resection of the disease cord, clostridial collagenase injection and needle aponeurotomy. These procedures differ in the amount of type-1 collagen-rich extra-cellular matrix remains *in situ *after treatment. While it is premature to translate the *in vitro *data reported here to treatment recommendations, it is possible that treatment that disrupt mechanical tension and deplete disease-associated collagen may have beneficial effects on disease progression and recurrence beyond mechanical disruption of the disease cord alone. In contrast, treatment that disrupt the DD cord and relieve mechanical tension but do not alter the level of disease-associated collagen within the fascia may allow DD-associated fibroblasts to maintain their disease phenotype, potentially resulting in higher rates of disease progression and recurrence.

## Conclusion

In summary, these findings implicate type-1 collagen as a regulator of DD cell phenotype and emphasize the importance of modeling fibroproliferative diseases in general, and DD in particular, in culture systems that include ECM components of the *in vivo *disease environment.

## Competing interests

The authors declare that they have no competing interests.

## Authors' contributions

LV performed all the western immunoblotting and immunofluorescence microscopy in this report. AN and YW performed the primary cell cultures and assisted LV in collagen preparation. DBO conceived of the study, and BSG participated in its design and coordination and helped to draft the manuscript. All authors read and approved the final manuscript.

## Pre-publication history

The pre-publication history for this paper can be accessed here:


